# Sialadenoma Papilliferum: Clinical Misdiagnosis with a Histological Decree

**DOI:** 10.1155/2012/356271

**Published:** 2012-03-19

**Authors:** A. Anuradha, V. V. S. Ram Prasad, Bina Kashyap, Vijay Srinivas

**Affiliations:** ^1^Department of Oral Pathology, Saint Joseph Dental College and Hospital, Duggirala, Eluru, 534004, India; ^2^Anuradha ENT Hospital, Eluru Road, Gudivada, Krishna 521301, India

## Abstract

Sialadenoma papilliferum is a rare salivary gland tumor clinically resembling papilloma originating probably from the excretory duct. It is characterized by a biphasic growth pattern of exophytic squamous component and endophytic glandular component. We report a rare case of sialadenoma papilliferum in the floor of the mouth with epithelial dysplasia with pertinent review of literature. The present case highlights the importance of keeping sialadenoma papilliferum as a differential diagnosis of exophytic papilliferous oral lesions and the need to explore the etiology and malignant potential of the tumor.

## 1. Introduction

Sialadenoma papilliferum (SP) is a rare, distinctive benign tumor of salivary gland classified under the ductal papillomas by WHO. Since the lesion was first described, less than 50 cases have been reported in literature. It was first described and named by Abrams and Finck [1] in 1969 and since then the histogenesis has been a topic of great controversy. The average age of occurrence is 59.2 years with a male predilection, commonly involving the minor salivary glands, with the lesions ranging 0.3–2 cm in size [2]. Most of the cases reported in literature are involving the palate with a clinical presentation as a well-circumscribed, exophytic papillary growth lacking a definite capsule. Histopathologically, the tumor shows surface papillary fronds and underlying tortuous dilated ducts. The present case has been reported for its rare site of occurrence in the floor of the mouth and the presence of dysplastic features in the epithelium lining the papillary fronds, which is likely the second case report in the literature.

## 2. Case Report

A 65-year-old male patient presented with a complaint of soreness in the anterior floor of the mouth, for one year. Clinically, the lesion was well circumscribed, white, and 1 cm diameter with a rough papilliferous surface. It was provisionally diagnosed as papilloma and excision of the lesion was done under local anesthesia. Grossly, the lesion was 0.8 cm with a rough verrucous surface.

**Figure 1:**
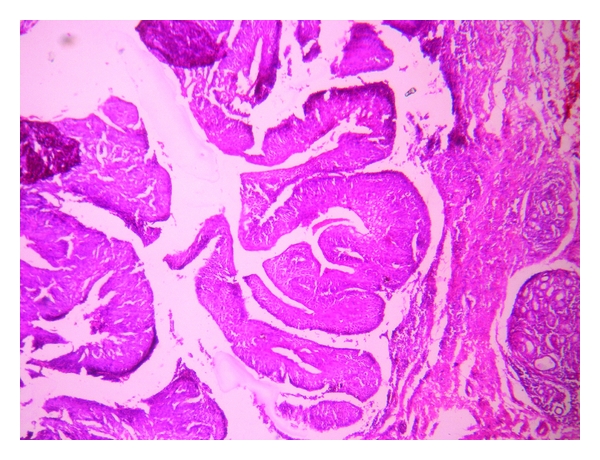
Surface papillary projections lined by a parakeratotic, acanthotic stratified squamous epithelium at 40x.

**Figure 2:**
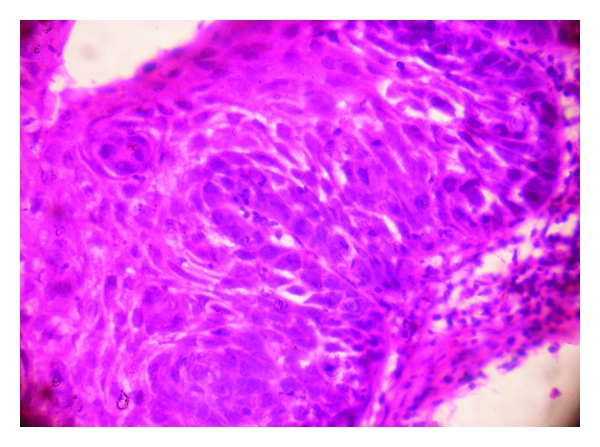
Dysplastic features such as basal cell hyperplasia, nuclear and cellular pleomorphism, and nuclear hyperchromatism at 400x.

**Figure 3:**
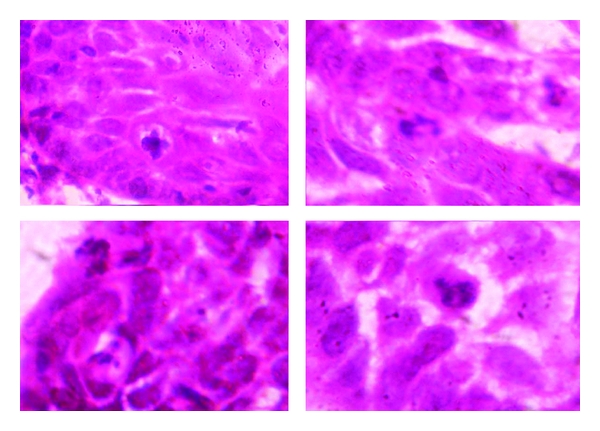
Mitotic figures under oil immersion 1000x.

**Figure 4:**
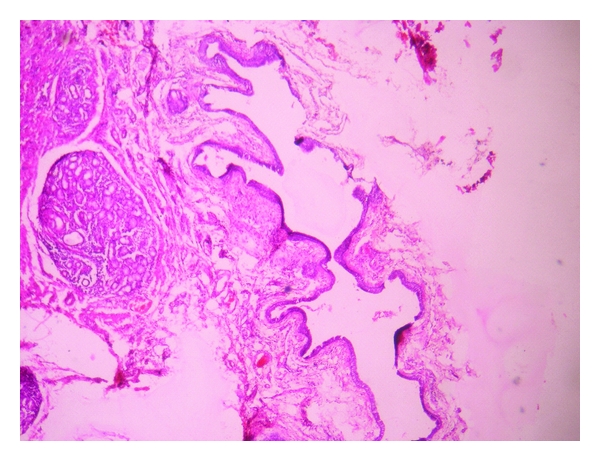
Mucous salivary gland with extralobular dilated tortuous ducts at 40x.

**Figure 5:**
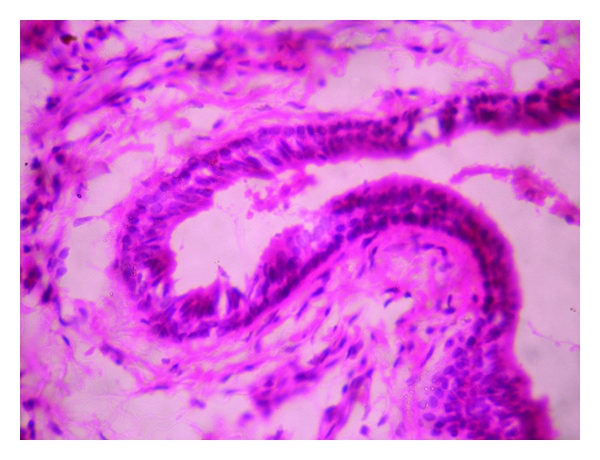
Dilated tortuous ducts lined by luminal columnar cells and abluminal cuboidal cells at 400x.

Histopathologically, the lesion showed exoendophytic proliferation of ductal epithelium with numerous papillary projections lined by a parakeratotic, acanthotic stratified squamous epithelium (Figure 1). The papillary epithelium showed dysplastic features such as basal cell hyperplasia, nuclear and cellular pleomorphism, nuclear hyperchromatism, and 3 to 4 mitotic figures per high power field (Figures 2 and 3). The papillae showed thin fibro-vascular cores with mild mixed inflammatory infiltrate. At the junction with normal mucosa, the epithelium showed wide, rounded rete pegs pushing into the connective tissue. Below the papillary proliferations, the connective tissue showed mucous salivary gland with extralobular dilated tortuous ducts lined by luminal columnar cells and abluminal cuboidal cells (Figures 4 and 5). Based on these features, it was diagnosed as sialadenoma papilliferum. Since the lesion was adequately, excised no additional therapy was given and the patient is on regular follow-up for the past one year without any recurrence.

## 3. Discussion

Sialadenoma papilliferum tabulated under the ductal papillomas accounts for less than 1% of all minor salivary glands tumors, and since its first description, very few cases are reported. Abrams and Finck [1] believe the lesion to originate from myoepithelium cells whereas Freedman and Lumerman [3] proposed it to originate from excretory duct reserve cells, since it can develop outside the salivary gland proper and can contain both mucous producing and squamous cells. Fantasia et al. [4] suggested that the tumor cells of SP are derived from excretory ducts. Some authors even considered it as a hyperplastic lesion based on the facts that it never exceeds 1 cm in size and the absence of encapsulation. Immunohistochemical study by Gomes et al. [5] shows a strong positivity of the tumor cells to cytokeratins CKs 7 and 8, indicating a more distal origin. Nakahata et al. [6] suggested its origin from a primitive precursor cells capable of multidirectional differentiation. The overall review of literature suggests a vague genesis of SP with its more likely origin from the excretory ducts.

Intraorally, SP presents as a painless papillary growth at the junction of hard and soft palate. The other sites are the buccal mucosa, retromolar pad, lips, faucial pillars, parotid gland, and very rarely floor of the mouth. A correct preoperative diagnosis is seldom made because of its papillary clinical presentation, rarity of the lesion, and its contrast clinical pattern with most intraoral salivary gland tumors which present as submucosal nodular swellings. It is confused with other papillary lesions like squamous papilloma, early or incipient verrucous carcinoma, inverted ductal papilloma, and warty dyskeratoma. The present case was also misdiagnosed clinically as a common squamous papilloma; hence photographs were not taken.

Histologically, the current case showed all pathognomic features of SP and in addition moderate dysplasia in the surface papillary epithelium was evident. Ponniah [7] recently reported sialadenoma papilliferum with dysplasia in surface epithelium ranging from moderate dysplasia to carcinoma in situ. Malignant analog of SP was proposed initially by Solomon et al. but after evaluating the histopathological features of his case it best be entitled as mucoepidermoid carcinoma [8]. Shimoda et al. [9] reported the only case of SP with an explicit malignant component showing areas of epithelial-myoepithelial carcinoma in the upper submucosa and high-grade carcinoma similar to micropapillary carcinoma of the breast, deeper in location without epithelial dysplasia in the exophytic component. Nasu et al. [8] rule out the notion of hyperplastic nature of SP and suggest it to embody a true neoplasm based on the proliferation of double-layered ductal epithelium to form intraductal papillary growth similar to papillary cystadenoma lymphomatosum. They suggested that the microscopic features of nuclei and cytoplasm of the tumor cells are somewhat different from those of simple hyperplastic duct cells in shape, site, and stainability. They also unseemly ascribed the overlying squamous epithelial proliferation to collateral hyperplasia from surrounding normal squamous epithelium. The presence of dysplasia in the surface epithelium in the current case and the one reported by Ponniah [7] rules out the likelihood of simple hyperplastic scenery of surface epithelium and validates the most plausible concept of a true neoplasm with biphasic growth pattern, both of which are neoplastic.

Though the clinical course of the lesion is benign, the presence of dysplasia in reality questions its malignant potential. The frequent incidence of inflammatory infiltrate underlying the exophytic component in almost all reported cases is also a point of great contemplation. The limited number of cases and studies on sialadenoma papilliferum prevents us from drawing any lasting conclusions regarding its etiology and its itinerary of malignant transformation.

## References

[1] Abrams AM, Finck FM (1969). Sialadenoma papilliferum. A previously unreported salivary gland tumor. *Cancer*.

[2] Brannon RB, Sciubba JJ, Giulani M (2001). Ductal papillomas of salivary gland origin: a report of 19 cases and a review of the literature. *Oral Surgery, Oral Medicine, Oral Pathology, Oral Radiology, and Endodontics*.

[3] Freedman PD, Lumerman H (1978). Sialadenoma papilliferum. *Oral Surgery Oral Medicine and Oral Pathology*.

[4] Fantasia JE, Nocco CE, Lally ET (1986). Ultrastructure of sialadenoma papilliferum. *Archives of Pathology and Laboratory Medicine*.

[5] Gomes APN, Sobral APV, Loducca SVL, de Araújo VC (2004). Sialadenoma papilliferum: immunohistochemical study. *International Journal of Oral and Maxillofacial Surgery*.

[6] Nakahata A, Deguchi H, Yanagaw A (1990). Coexpression of immediate sized filaments in Sialadenoma Papilliferum and other salivary gland neoplasm. *Journal of Oral Pathology & Medicine*.

[7] Ponniah I (2007). A rare case of sialadenoma papilliferum with epithelial dysplasia and carcinoma in situ. *Oral Surgery, Oral Medicine, Oral Pathology, Oral Radiology and Endodontology*.

[8] Nasu M, Takagi M, Ishikawa G (1981). Sialadenoma papilliferum: report of case. *Journal of Oral Surgery*.

[9] Shimoda M, Kameyama K, Morinaga S (2004). Malignant transformation of sialadenoma papilliferum of the palate: a case report. *Virchows Archiv*.

